# Nε-(1-Carboxymethyl)-L-lysine/RAGE Signaling Drives Metastasis and Cancer Stemness through ERK/NFκB axis in Osteosarcoma

**DOI:** 10.7150/ijbs.90817

**Published:** 2024-01-12

**Authors:** Ting-Yu Chang, Kuo-Cheng Lan, Chia-Hung Wu, Meei-Ling Sheu, Rong-Sen Yang, Shing-Hwa Liu

**Affiliations:** 1 Graduate Institute of Toxicology, College of Medicine, National Taiwan University, Taipei, Taiwan.; 2 Department of Emergency Medicine, Tri-Service General Hospital, National Defense Medical Center, Taipei, Taiwan.; 3 Institute of Biomedical Sciences, National Chung Hsing University, Taichung, Taiwan.; 4 Department of Medical Research, Taichung Veterans General Hospital, Taichung, Taiwan.; 5 Department of Orthopedics, National Taiwan University Hospital, Taipei, Taiwan.; 6 Department of Medical Research, China Medical University Hospital, China Medical University, Taichung, Taiwan.; 7 Department of Pediatrics, College of Medicine, National Taiwan University & Hospital, Taipei, Taiwan.

**Keywords:** osteosarcoma, Nε-(1-Carboxymethyl)-L-lysine, cancer stemness, metastasis

## Abstract

Osteosarcoma is an extremely aggressive bone cancer with poor prognosis. Nε-(1-Carboxymethyl)-L-lysine (CML), an advanced glycation end product (AGE), can link to cancer progression, tumorigenesis and metastasis, although the underlying mechanism remains unclear. The role of CML in osteosarcoma progression is still unclear. We hypothesized that CML could promote migration, invasion, and stemness in osteosarcoma cells. CML and its receptor (RAGE; receptor for AGE) were higher expressed at advanced stages in human osteosarcoma tissues. In mouse models, which streptozotocin was administered to induce CML accumulation in the body, the subcutaneous tumor growth was not affected, but the tumor metastasis using tail vein injection model was enhanced. In cell models (MG63 and U2OS cells), CML enhanced tumor sphere formation and acquisition of cancer stem cell characteristics, induced migration and invasion abilities, as well as triggered the epithelial-mesenchymal transition process, which were associated with RAGE expression and activation of downstream signaling pathways, especially the ERK/NFκB pathway. RAGE inhibition elicited CML-induced cell migration, invasion, and stemness through RAGE-mediated ERK/NFκB pathway. These results revealed a crucial role for CML in driving stemness and metastasis in osteosarcoma. These findings uncover a potential CML/RAGE connection and mechanism to osteosarcoma progression and set the stage for further investigation.

## Introduction

Osteosarcoma is a rare and aggressive type of bone cancer that typically affects children and young adults 1. Despite advances in treatment, the prognosis for patients with osteosarcoma remains poor, with high rates of recurrence and metastasis 2. The incidence of osteosarcoma is estimated to be around 2.3 cases per million people per year, and the overall 5-year survival rate for osteosarcoma is approximately 65% 3. The 5-year survival rate for localized osteosarcoma is approximately 70-80%, while the 5-year survival rate for metastatic osteosarcoma is approximately 20-30% 4. Osteosarcoma cells are highly mobile and have ability to invade and spread to other organs and tissues, such as the lungs, making it difficult to treat 5.

One of the reasons for the poor prognosis of osteosarcoma is the presence of cancer stem cells (CSCs) within the tumor. CSCs are a subpopulation of cells within a tumor that have possess stem cell-like properties, such as self-renewal and differentiation, and are thought to be responsible for tumor growth 6, metastasis 7, and drug resistance 8. In osteosarcoma, CSCs have been shown to interact with the tumor microenvironment, which includes other cell types, extracellular matrix components, and soluble factors 9-12, contributing to tumor growth and metastasis. Additionally, several studies have suggested that targeting CSCs could be a promising strategy to prevent or inhibit metastasis in osteosarcoma 13. These studies have identified potential molecular targets that are specifically expressed in CSCs and that could be used to develop targeted therapies for osteosarcoma metastasis. A number of stemness markers or genes that are associated with pluripotency, self-renewal, and differentiation, such as CD133, CD44, CD117, ALDH, Oct4, and Sox2 14-19 of osteosarcoma. Recent studies have shown that osteosarcoma CSCs are regulated by several signaling pathways, including Wnt/β-catenin, Notch, Hedgehog, and PI3K/Akt/mTOR pathways 20-23. These pathways are involved in various cellular processes, such as cell proliferation, differentiation, apoptosis, and migration, and are frequently dysregulated in cancer. Overall, understanding the biology of osteosarcoma CSCs is essential for developing more effective therapies that target these cells and improve the outcomes of osteosarcoma patients.

Advanced glycation end products (AGEs) are compounds that can be formed by the non-enzymatic reaction between reducing sugars and amino acids, particularly lysine 24. One of these AGEs, Nε-(1-carboxymethyl)-L-lysine (CML), not only forms under physiological conditions, but also forms in various foods cooked at high temperatures, such as meat, fish, and dairy products, as well as in tobacco smoke 25. CML has been implicated in age-related diseases, such as diabetes, atherosclerosis, and Alzheimer's disease, by promoting oxidative stress, inflammation, and cellular damage 26-28. Furthermore, CML may promote cancer cell proliferation, invasion, and survival by binding to and activating the receptor for AGEs (RAGE) signaling 29, 30. RAGE is a transmembrane receptor that is overexpressed in several types of cancer, such as breast, lung, pancreatic, colon cancer, and osteosarcoma 31-35. Activation of RAGE and its ligands (including AGEs) in cancer cells promotes cytokine and growth factor production, tumor growth, angiogenesis, and survival, triggers various signaling pathways and is involved in metastasis 36-39. Targeting RAGE through inhibition of expression or activity has been shown to promise in preclinical models to decrease tumor growth, angiogenesis, and metastasis 40-42. However, the relationship between CML and osteosarcoma is complex and requires a full understanding of the mechanisms by which CML targets osteosarcoma.

In this study, we sought to investigate the impacts of CML on migration, invasion, and stemness of osteosarcoma and the molecular mechanism involved. Our findings suggest that CML increases the cancer stemness and migration and invasion abilities of osteosarcoma cells by activating the RAGE-NFκB signaling pathway. Moreover, this study indicates a possible link between CML accumulation and osteosarcoma progression through CML-induced stemness and metastasis of osteosarcoma cells. Understanding the role of CML in osteosarcoma progression and its potential as a therapeutic target may help improve the prognosis for patients with this disease.

## Materials and Methods

### Human Tissue Microarray (TMA) and immunohistochemistry (IHC) staining

The human osteosarcoma tissue microarray OS804d was purchased from US, Biomax Inc. containing 40 cases of osteosarcoma. We performed IHC staining using a commercial IHC kit (cat. no. ab64264, Abcam, Cambridge, U.K.), following the manufacturer's procedural guidelines. Tissue sections were removed the wax using Sub-X and then rehydrated with a series of ethanol solutions. Antigen retrieval was performed by incubating slides with protease, and then blocking with protein block reagent for 10 minutes. The CML (cat. no. ab125145, Abcam) and RAGE (cat. no. ab3611, Abcam) antibodies were incubated with the sections overnight at 4°C. HRP-conjugated secondary antibody was then applied, and the sections were treated with DAB substrate. Subsequently, the slides were counterstained with hematoxylin, and sealed with mounting medium. The stained sections were analyzed using the IHC profiler plugin in ImageJ software. A score of 0 indicated negative staining, score of 1 indicated low positive staining, score of 2 indicated positive staining, and score of 3 indicated high positive staining.

### Animal xenograft experiments

All animal experiments conducted in this study adhered to the guidelines set by the Association for Assessment and Accreditation of Laboratory Animal Care International and were approved by the Institutional Animal Care and Use Committee at the National Taiwan University College of Medicine (Taipei, Taiwan) (IACUC No. 20220101). Male NOD/SCID mice (*n* = 7/group) aged 5-6 weeks were procured from BioLASCO (Taipei, Taiwan). Hyperglycemia with high AGEs levels was induced in mice by intraperitoneal injection of streptozotocin (STZ, Sigma Aldrich; 40 mg/kg), dissolved in citrate buffer, for five consecutive days 43. Mice with blood glucose levels exceeding 300 mg/dL after 14 days were labeled as the hyperglycemic group. Subsequently, 1 × 10^6^ human osteosarcoma MG63 cells were subcutaneously implanted into the dorsal flanks of mice for tumor growth. Tumor size was checked twice weekly and calculated the tumor volume using the formula [(length × width × width)/2]. After 28 days, mice were euthanized and their tumors were dissected for further analysis. For the *in vivo* extravasation assay, 1 × 10^6^ MG63 cells suspended in 100 μL PBS were injected into the tail vein of mice, with or without STZ treatment. We selected MG63 cells due to their established metastatic potential. Upon injection into the tail vein, the cells circulated through the bloodstream and primarily targeted pulmonary metastasis. After eight weeks post-injection, mice were humanely euthanized under anesthesia using a mixture of isoflurane and 3% oxygen, followed by cervical dislocation. The lungs tissues were subsequently excised for additional examination and analysis 44.

### Cell Cultures and Reagents

MG63 and U2OS human osteosarcoma cell lines were purchased from American Type Culture Collection (ATCC). MG63 cells were maintained in MEM medium (Gibco), and U2OS cells were grown in McCoy's 5A medium (Gibco) supplemented with 10% fetal bovine serum and 1% penicillin-streptomycin-amphotericin B solution (Sartorius). CML was obtained from Cayman Chemical and dissolved in PBS. Cells were treated with CML at concentrations of 25 μM and 50 μM for 6 days, with media changed every two days. In some experiments, RAGE expression was blocked with RAGE neutralizing antibody (cat. no. AGF1145; R&D systems) by pre-treatment for 2 hours.

### Transwell migration and invasion assay

To assess the migration and invasion capabilities of cells, we performed migration assays using Transwell inserts with a pore size of 8 µm (Corning Costar). For invasion assays, Transwell inserts were incubated with Matrigel matrix at 37°C for 1 h to allow the Matrigel matrix to create a gel. After that, around 8 × 10^4^ cells (for migration) or 1 × 10^5^ cells (for invasion) were placed in the upper chamber with serum-free medium. The lower chamber of the 24-well plate received medium with serum and was incubated for 16 h (for migration) or 24 h (for invasion). The discrepancy in incubation times between the migration assay (16 h) and invasion assay (24 h) is intentional and is based on established protocols in the literature 45. The longer incubation period for the invasion assay is commonly employed to allow for the more complex process of cell penetration through a matrigel or similar barrier. After incubation, cells on the upper membrane surface were carried out using a cotton swab, and cells that exhibited migration or invasion through the membrane were fixed with 4% paraformaldehyde for 30 min and subjected to staining with 0.05% crystal violet. The stained cells were imaged using a microscope, and the number of cells that had migrated or invaded through the membrane was quantified using ImageJ software.

### Western blot analysis and antibodies

Cells were harvested and lysed with RIPA lysis buffer containing protease cocktail inhibitor (cat. no. 78430, Thermo Fisher Scientific) overnight at -20℃, followed by high-speed centrifugation for 30 min. The supernatant and an equal amount of lysate protein were mixed in sample buffer and heated at 95°C for 10 min. The protein samples were loaded into the wells of a stacking gel and run SDS-PAGE, then transferred the proteins to PVDF membranes. Subsequent to this transfer, the membranes underwent blocking using 5% milk and were subjected to incubation with designated primary and secondary antibodies. The immunoreactive proteins were visualized using a chemiluminescence detection system (Bio-Rad). The primary antibodies were used for Western blot analysis: MRP1 (#72202), MDR1 (#13978), CD44 (# 37259), p-NFκB p65 (#3033), p-AKT (Ser473, #9271), AKT (#9272), p-GSK3α/β (Ser21/9, #9331), GSK3α/β (#5676), p-ERK1/2 (Tyr202/204, #9101), ERK1/2 (#9102) from Cell Signaling Technology. ALDH1A1 (GTX123973) from GeneTex. NANOG (ab80892) from Abcam. E-cadherin (sc-7870), NFκB p65 (sc-8008), β-actin (sc-47778) from Santa Cruz Biotechnology. Fibronectin (cat. no. 610077) from BD Transduction Laboratories. RAGE (cat. no. MAB5328) from Sigma-Aldrich.

### Sphere formation assay

Cells (1 × 10^3^) were seeded onto ultra-low attachment 24-well plates (Corning Costar) and incubated for 14-21 days. The sphere formation medium consisted of serum-free culture medium, B-27 supplement, recombinant human epidermal growth factor (EGF; 20 ng/ml), and basic fibroblast growth factor (FGF; 10 ng/ml). Sphere was monitored regularly and the number of spheres with a diameter of at least 50 µm was recorded.

### Statistical Analysis

The data were presented as the mean ± S.D. derived from a minimum of three independent experiments. A one-way ANOVA with Tukey's post hoc analysis was conducted using GraphPad Prism 8 software to determine the significant difference among different groups, and the significance level was set at *p* < 0.05.

## Results

### CML and RAGE contribute to the progression and metastasis of osteosarcoma

**Figure 1:**
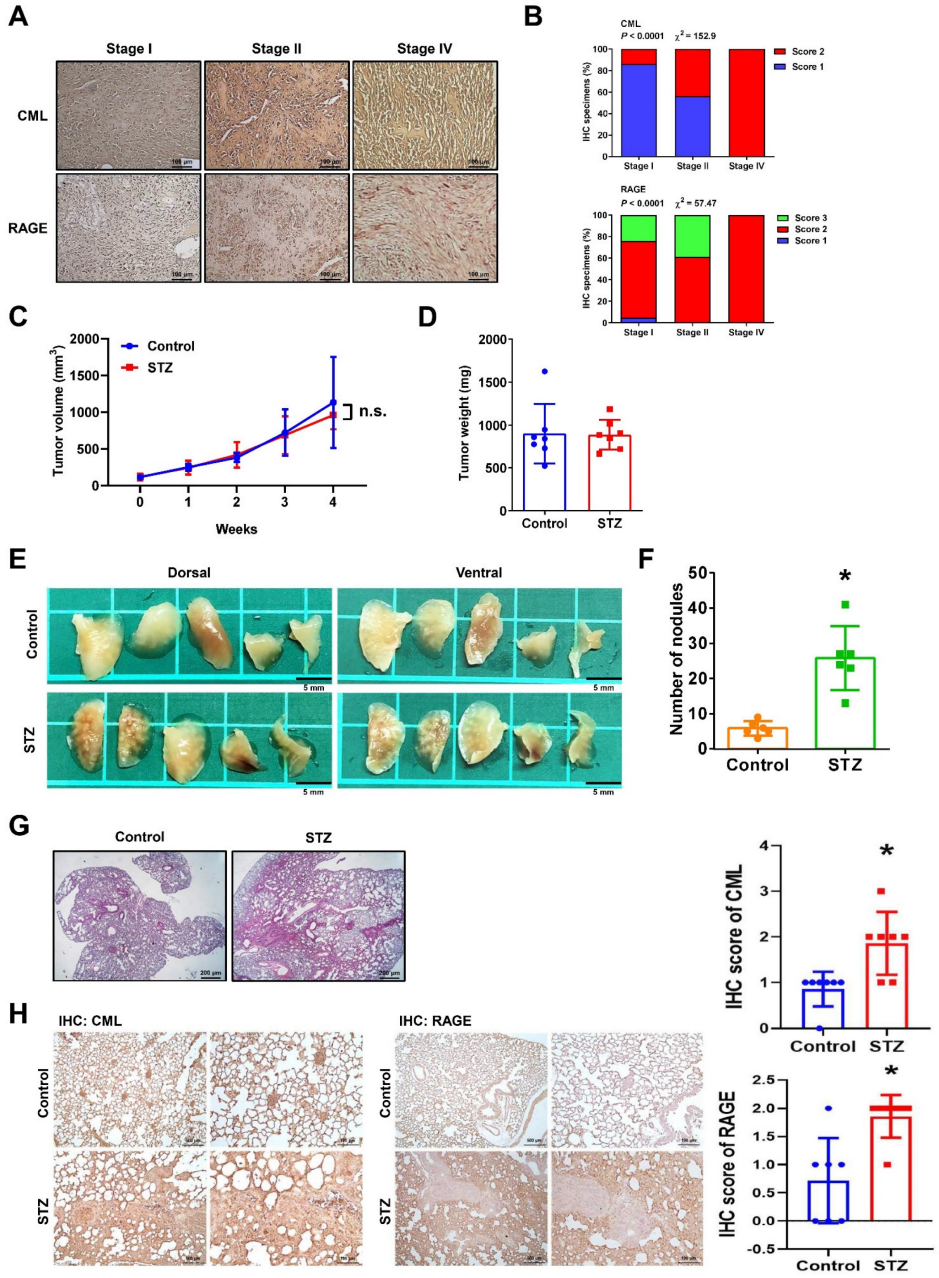
CML accumulation increase tumor metastasis but does not affect primary tumor growth. (**A**) CML and RAGE IHC staining in human osteosarcoma tissue microarray (*n* = 40) with different stages were shown. Scale bar, 100 μm. (**B**) Quantification of CML and RAGE for IHC staining was presented as a percentage. The significant difference was analyzed using Chi-square analysis followed by Fisher's exact test. (**C, D**) MG63 cells were injected subcutaneously into the dorsal flanks of mice with or without prior treatment with STZ (*n* = 7 per group). The tumor growth was measured weekly, and the tumor weight was recorded after euthanizing. (**E, F**) MG63 cells were intravenously injected in mice with or without pretreatment with STZ. The metastatic tumor nodules in the lungs were examined and quantified. *n* = 6 per group. *, *p* < 0.05. (**G**) Representative H&E staining images of the lungs were shown. The tumor nodules were visualized as dark purple areas. Scale bar, 200 μm. (**H**) CML and RAGE IHC staining in the lungs of mice with or without STZ treatment (*n* = 7 per group). Scale bar, 500 μm (left panels), 100 μm (right panels). *, *p* < 0.05.

The CML and RAGE expression levels in human osteosarcoma were firstly investigated. An analysis of human tissue microarrays derived from osteosarcoma patient specimens using IHC staining was conducted. The clinical characteristics of the osteosarcoma patients included in the tissue microarray are detailed in **Table 1**. The results revealed a significant association between higher levels of CML and RAGE expression and more advanced tumor stages (**Figure 1A, 1B**). CML and RAGE are known to play roles in cancer progression and metastasis, with their expression levels having been reported to be upregulated under hyperglycemic conditions 29, 33, 37. To investigate the impact of CML accumulation on primary tumor growth, we induced hyperglycemia in mice through intraperitoneal STZ treatment to induce hyperglycemia with high CML, followed by the subcutaneous injection of MG63 cells into the dorsal flank. Both blood glucose and serum CML levels significantly increased in the STZ group compared to the control groups (glucose: STZ group: 502.3 ± 31.6, control group, 122.1 ± 26.6 mg/dL; serum CML: STZ group, 3.38 ± 2.09, control group, 1.85 ± 0.52 μg/mL; n = 7, *p* < 0.05). The results indicated that there was no significant difference in the size or growth rate of primary tumors between the hyperglycemic and control groups. These results suggest that hyperglycemia with high serum CML induced by STZ treatment does not directly influence primary tumor growth in this animal model (**Figure 1C, 1D**). However, a noteworthy observation emerged in a mouse metastasis model when the lung tissues of STZ-treated mice injected with MG63 cells into the tail vein were further examined. The increased number of nodules in the lung tissues of STZ-treated mice were observed (**Figure 1E, 1F**). Furthermore, consistent with the increased nodules in the lung tissues of STZ-treated mice, the histological analysis using H&E staining revealed a significant enlargement in the area of metastasis in the lungs of STZ-treated group compared to the control group (**Figure 1G**). As shown in **Figure 1H,** the results from IHC staining indicated that the expression levels of CML and RAGE in the lungs of STZ group are significantly higher than those in the control group. These results suggest that the increased CML/RAGE signals may enhance the metastatic potential of osteosarcoma cells *in vivo*.

### CML enhances cell migration, invasion, EMT process, tumor sphere formation, and cancer stem cell properties in osteosarcoma cells

**Figure 2:**
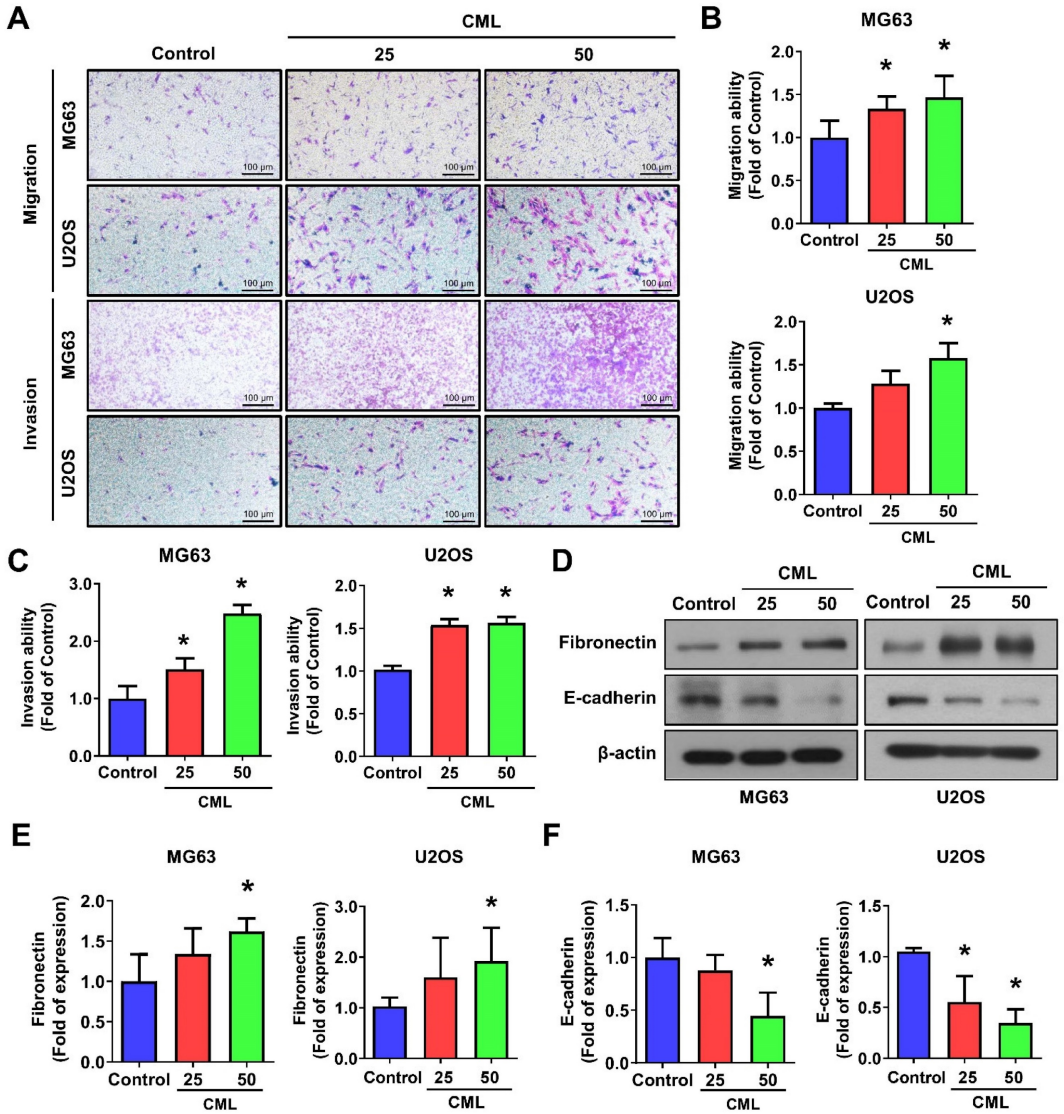
CML enhances the migration, invasion and expression of EMT-related markers. (**A, B, C**) Both cell migration and invasion abilities were induced with CML treatment at concentrations of 25 μM and 50 μM in MG63 and U2OS cells. Scale bar, 100 μm. (**D, E, F**) The protein expression of Fibronectin and E-cadherin were analyzed by Western blot and quantified. *, *p* < 0.05.

To assess the impact of CML on osteosarcoma cells, we conducted experiments using human osteosarcoma MG63 and U2OS cell lines. The significant increases in both migration and invasion capabilities in the CML (25 and 50 μM)-treated cells were observed (**Figure 2A-C**). The protein expression of epithelial-mesenchymal transition (EMT) markers was further examined. CML treatment led to the upregulation of Fibronectin expression (**Figure 2D, 2E**), concomitant with the downregulation of E-cadherin (**Figure 2D, 2F**). These results collectively indicated that CML has the potential to enhance the migration and invasion abilities of osteosarcoma cells while inducing the EMT process.

**Figure 3:**
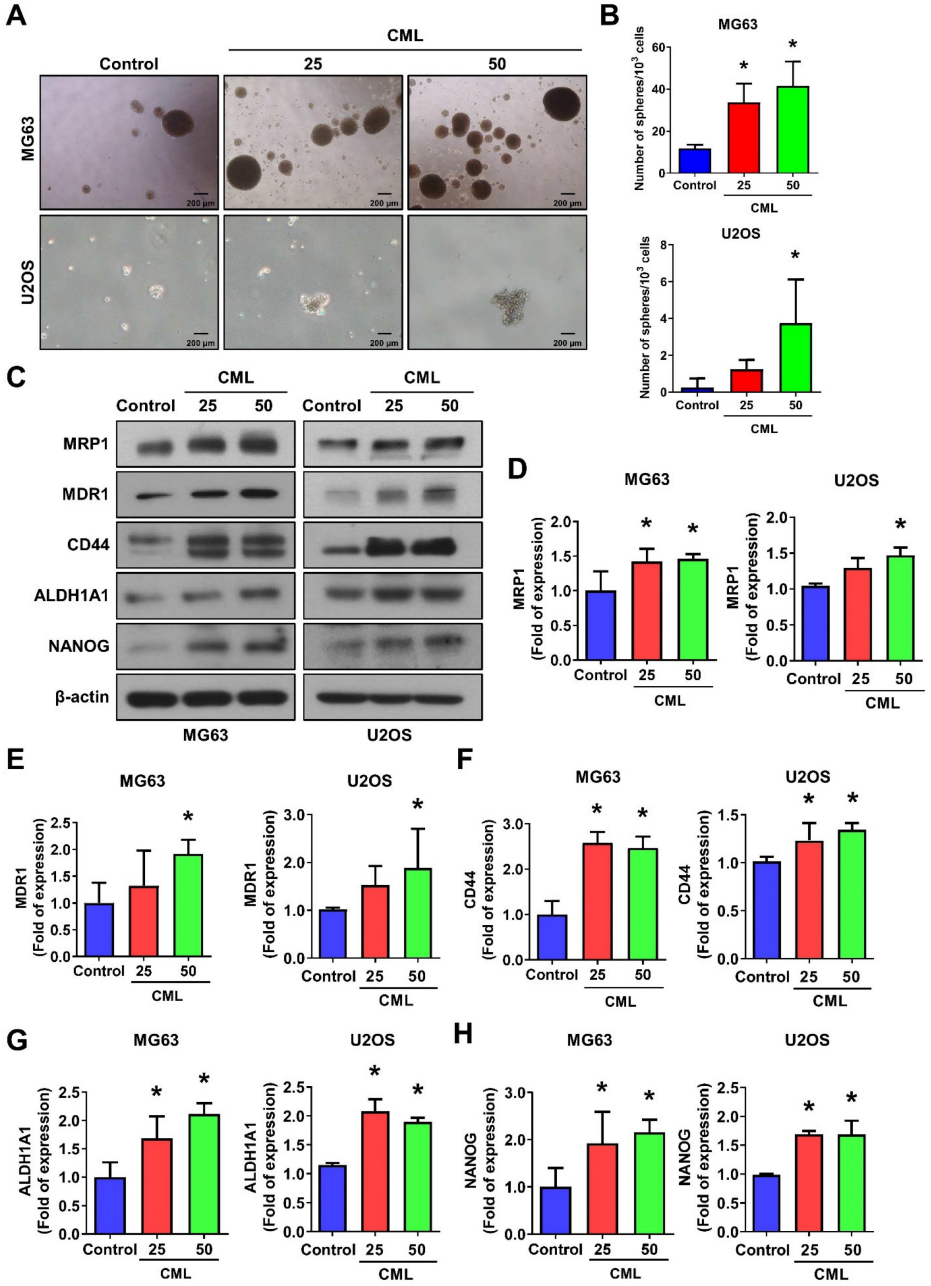
CML induces the sphere formation and stemness markers expression of osteosarcoma cells. (**A, B**) Self-renewal activity was determined by using the sphere formation assay in the presence or absence of CML treatment in MG63 and U2OS cells. The number of spheres was shown in **B**. Scale bar, 200 μm. (**C-H**) The protein expression of stemness markers, including MRP1, MDR1, CD44, ALDH1A1, and NANOG was determined and quantified in the indicated cells. *, *p* < 0.05.

Recent research has highlighted the pivotal role of cancer stem cells (CSCs) in initiating and driving the progression of osteosarcoma, influencing tumor growth, metastasis, and drug resistance 9-12. We sought to investigate the impact of CML treatment on the self-renewal capacity of these CSCs. To assess this, we performed a sphere formation assay, revealing a significant and dose-dependent increase in the number of spheres formed following CML (25 and 50 μM) treatment (**Figure 3A, 3B**). The difference in sphere formation between MG63 and U2OS cells may be attributed to the intrinsic characteristics of each cell line, like stemness or clonogenic potential. The size of the spheres directly reflects the proliferation of sphere-forming cells. At present, the most common methods for measuring these tumor spheres are to count their number or to assess the efficiency of spheres formation. This observation underscores the potential of CML to augment the self-renewal ability of CSCs. CSCs are known for their ability to evade treatment, often attributed to the overexpression of ATP-binding cassette (ABC) multidrug efflux transporters, including MDR1/ABCB1, BRCP1/ABCG2, and ABCB5 10, 15. We further found that CML treatment led to an increased protein expression of MRP1 and MDR1 in both MG63 and U2OS cells (**Figure 3C-E**). Additionally, CD44, a transmembrane glycoprotein associated with cancer stemness, exhibited a significant induction in its expression level upon CML treatment (**Figure 3C, 3F**).

A significant upregulation of ALDH1A1 protein, which is associated with cancer stemness and metastatic potential 46, was also observed in the CML-treated group compared to the control group (**Figure 3C, 3G**). Furthermore, we noted an increase in the protein expression of the stem-like gene NANOG following CML treatment (**Figure 3C, 3H**). Collectively, these findings provided evidence that CML treatment enhanced the self-renewal ability of CSCs and upregulated the protein expression associated with CSC properties. These results suggest that CML may contribute to tumor growth, metastasis, and the promotion of a cancer stem cell phenotype in osteosarcoma.

### CML induces RAGE and ERK/NFκB activation but not AKT in osteosarcoma cells

**Figure 4:**
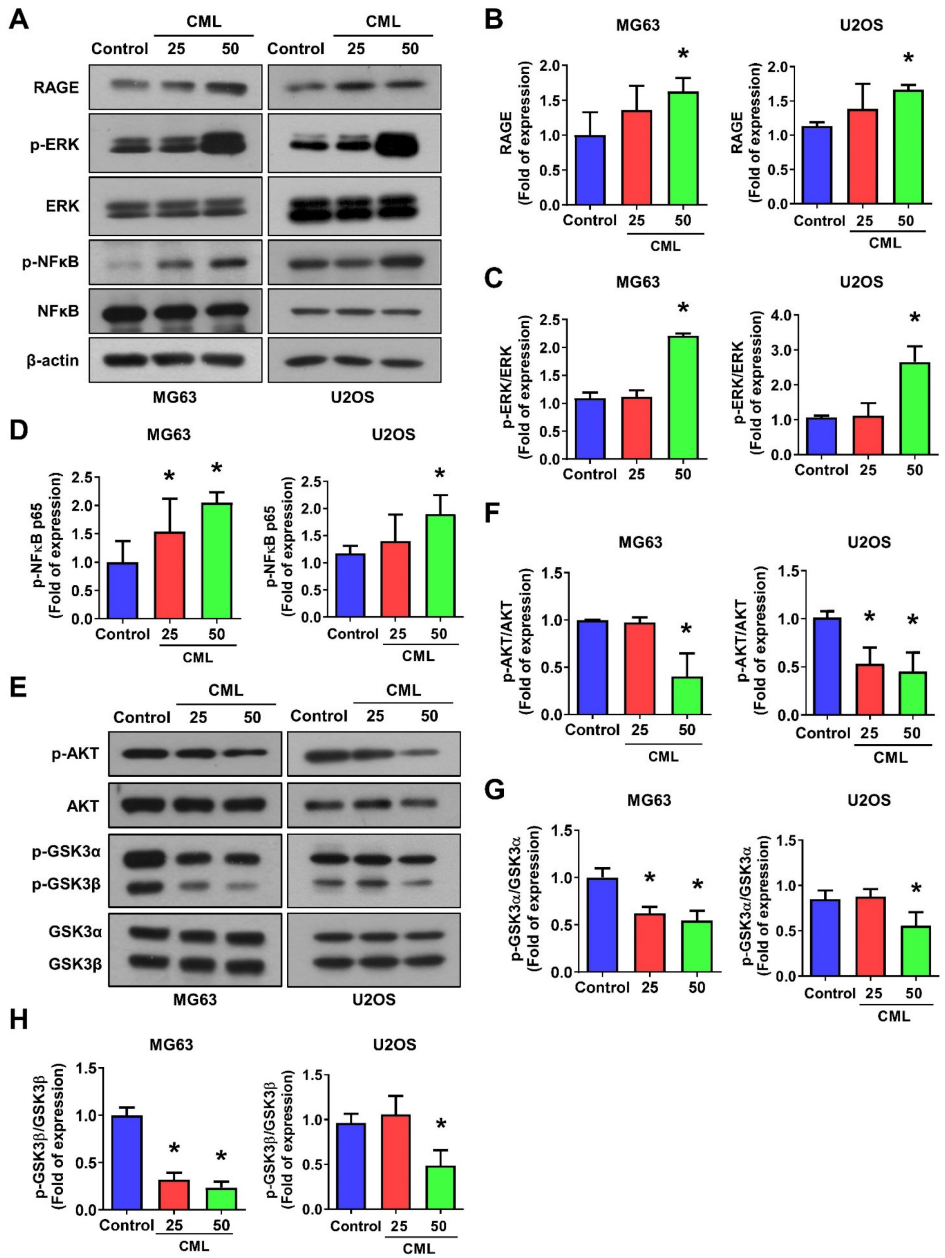
CML activates the expression of RAGE, ERK and NFκB, but inhibits the activation of AKT in osteosarcoma cells. (**A-D**) The protein expression of RAGE, p-ERK and p-NFκB was examined and quantified in the indicated cells. (**E-H**) The expression level of p-AKT, AKT, p-GSK3α/β and GSK3α/β with CML treatment in osteosarcoma cells were determined by Western blot and quantified. *, *p* < 0.05.

To delve into the underlying mechanism of CML/RAGE signaling in osteosarcoma cells, we conducted a comprehensive analysis of RAGE expression and the activation of downstream signaling pathways following CML treatment. First, we confirmed the expression of RAGE in osteosarcoma cells using Western blot analysis, revealing a notable upregulation of RAGE protein expression in response to CML treatment (**Figure 4A, 4B**). Previous study has provided valuable insights into RAGE-mediated signaling. Notably, it has been demonstrated that RAGE activates the ERK and NFκB signaling pathways, thereby influencing crucial cellular processes, such as proliferation, differentiation, and survival 47. We further explored the activation of ERK and NFκB signals in CML-treated osteosarcoma cells. The results showed a significant induction in the phosphorylation of both ERK and NFκB p65 in cells treated with CML compared to the control group (**Figure 4A, 4C, 4D**). We next assessed the activation of AKT and GSK3α/β, which signaling pathways intricately linked to cell survival and proliferation. Intriguingly, our results demonstrated that CML treatment in osteosarcoma cells led to a reduction in the phosphorylation of both AKT and GSK3α/β (**Figure 4E-H**). These findings suggest that CML treatment upregulates the RAGE/ERK/NFкB signals while concurrently downregulating the activation of AKT and GSK3α/β in osteosarcoma cells.

### RAGE is involved in CML-induced ERK and NFкB signaling, cell mobility, and CSCs properties in osteosarcoma cells

**Figure 5:**
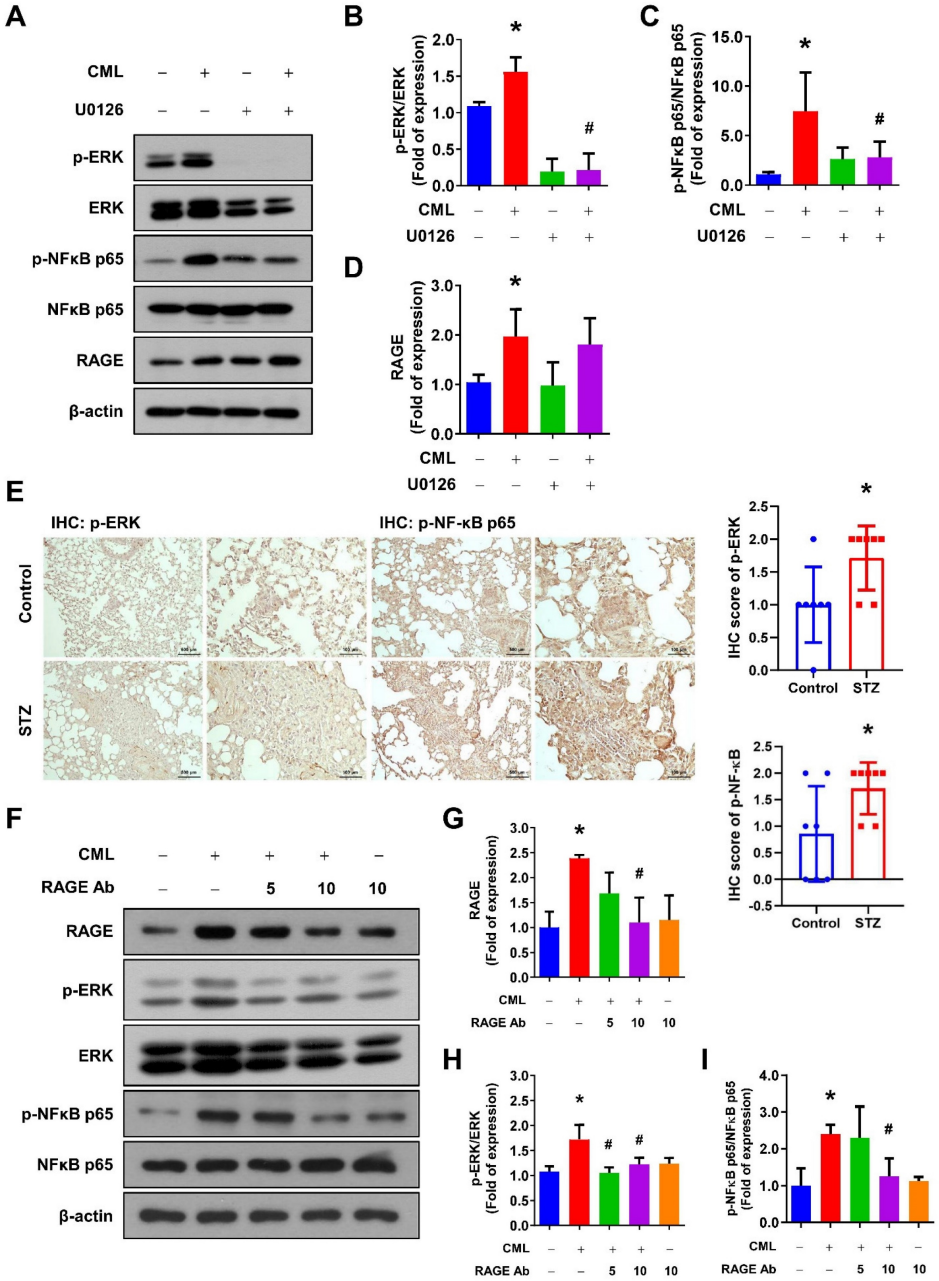
Blocking of ERK by U0126 and RAGE by anti-RAGE antibody attenuates the CML-induced NFκB activation. (**A-D**) The protein expression level of p-ERK, ERK, pNFκB, NFκB and RAGE, were examined and quantified with pretreatment with ERK inhibitor U0126 (20 μM) for 1 h in the presence or absence of CML (50 μM) treatment. *, *p* < 0.05 as compared to the Control group. #, *p* < 0.05 as compared to the CML group. (**E**) p-ERK and p-NFκB-p65 IHC staining in the lungs of mice with or without STZ treatment (*n* = 7 per group). Scale bar, 500 μm (left panels), 100 μm (right panels). *, *p* < 0.05. (**F-I**) The protein expression level of p-ERK, ERK, p-NFκB, NFκB and RAGE were examined and quantified with pretreatment with anti-RAGE antibody for 2 h in the presence or absence of CML (50 μM) treatment. *, *p* < 0.05 as compared to the Control group. #, *p* < 0.05 as compared to the CML group.

To further examine the involvement of the ERK signaling pathways in CML/RAGE signaling axis, we assessed the phosphorylation of ERK and NFκB following treatment with the ERK inhibitor U0126. As shown in **Figure 5A-D**, the ERK inhibitor effectively and completely suppressed the phosphorylation of ERK and NFκB induced by CML treatment, highlighting the critical role of ERK in this signaling cascade. Furthermore, we have investigated the activation status of ERK and NFκB-p65 in the lung tissues of mice utilizing IHC staining. Our findings revealed a significant elevation in the phosphorylation levels of p-ERK and p-NFκB in the lungs of the STZ group compared to those in the control group (**Figure 5E**). We also conducted experiments in which the cells were pre-treated with a RAGE neutralizing antibody prior to CML treatment. Strikingly, the activation of both ERK and NFκB was entirely abolished in the presence of the anti-RAGE antibody (**Figure 5F-I**). These results underscored the essential role of RAGE in facilitating CML-mediated activation of the ERK and NFκB signaling pathways.

**Figure 6:**
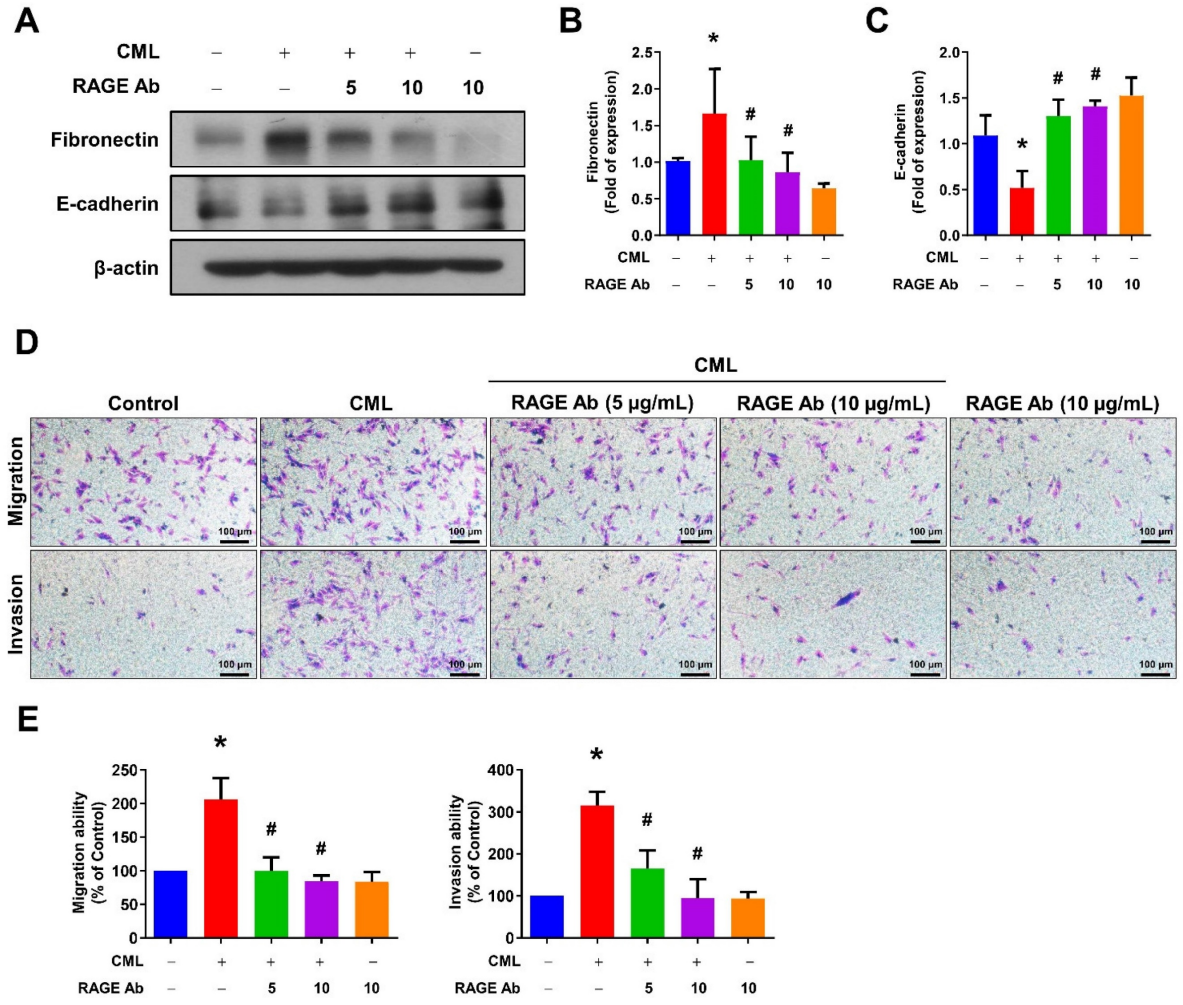
Inhibition of RAGE reduced the process of EMT and cell mobility. (**A-C**) The protein expression of Fibronectin and E-cadherin were analyzed in the indicated cells by Western blot and quantified. (**D, E**) The migration and invasion abilities were examined with pretreatment with anti-RAGE antibody for 2 h in the presence or absence of CML (50 μM) treatment. *, *p* < 0.05 as compared to the Control group. #, *p* < 0.05 as compared to the CML group.

**Figure 7:**
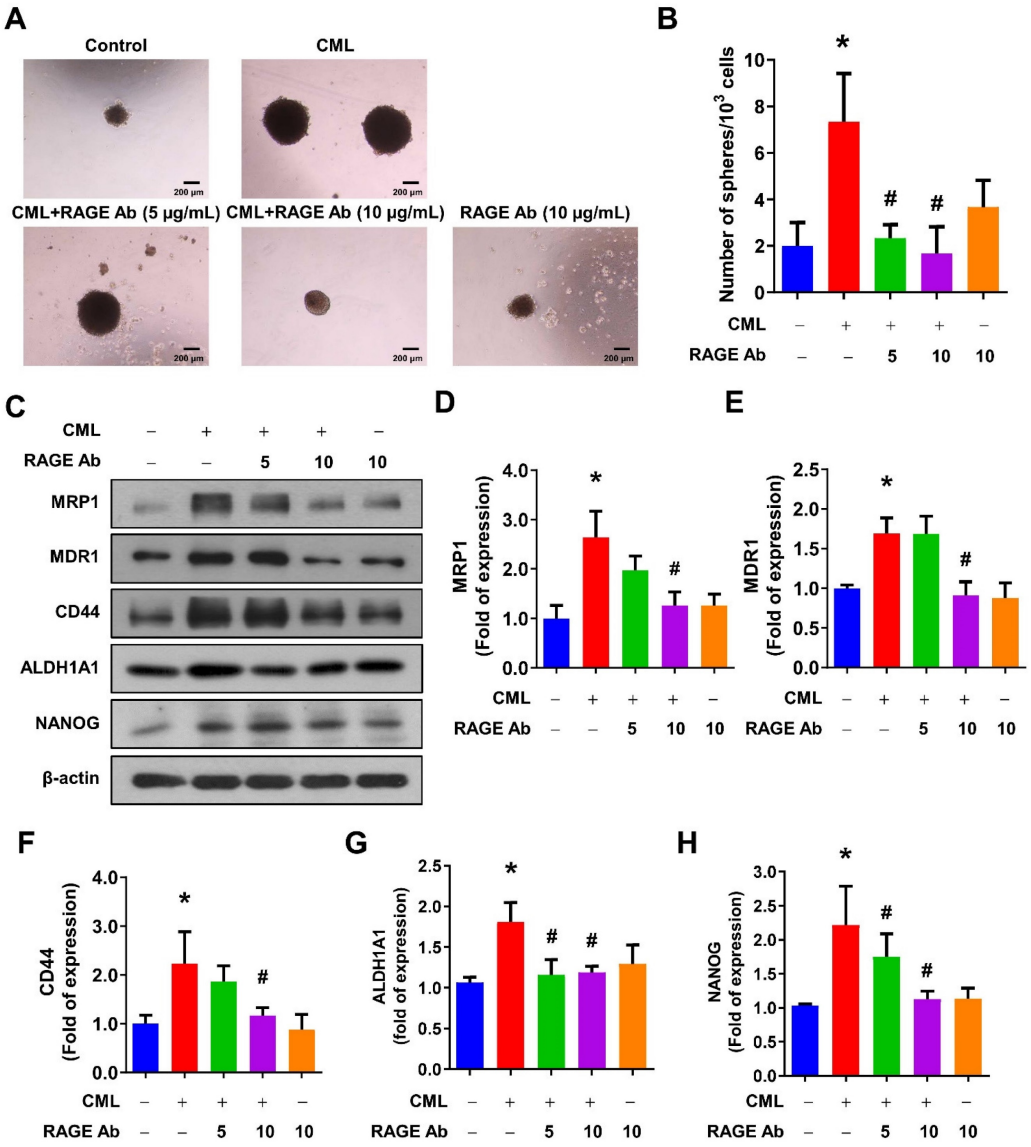
Inhibition of RAGE reduced the CSC stemness. (**A, B**) The sphere formation abilities were shown and quantified with pretreatment with anti-RAGE antibody for 2 h in the presence or absence of CML (50 μM) treatment. *, *p* < 0.05 as compared to the Control group. #, *p* < 0.05 as compared to the CML group. (**C-H**) The protein expression of stemness markers, including MRP1, MDR1, CD44, ALDH1A1, and NANOG was determined and quantified in the indicated cells. *, *p* < 0.05 as compared to the Control group. #, *p* < 0.05 as compared to the CML group.

**Figure 8:**
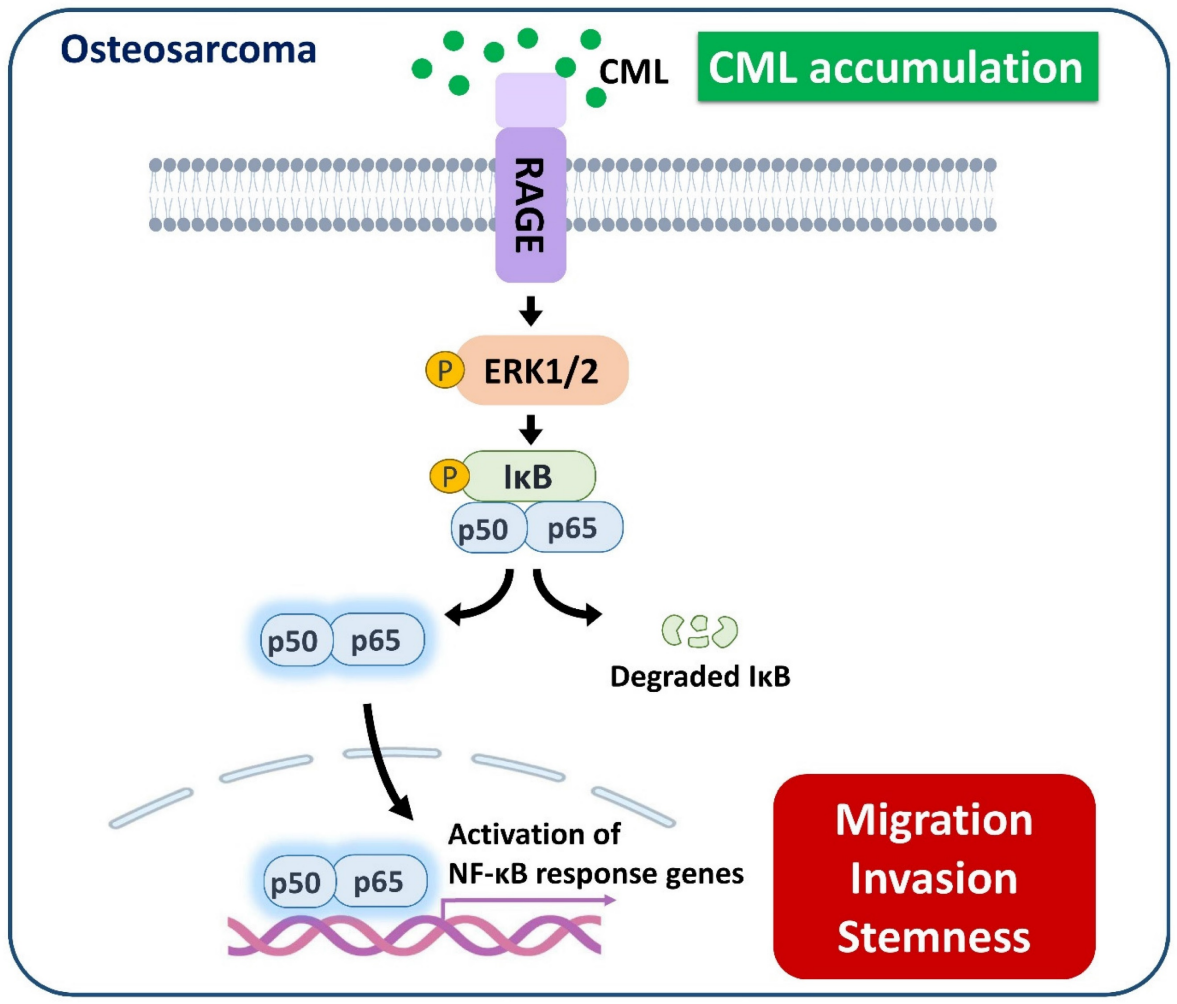
** A model of the possibly regulatory mechanism of CML in osteosarcoma.** The application of CML triggers migration, invasion and stemness, while concurrently activating RAGE/ERK/NFκB signaling.

We sought to elucidate the role of RAGE in mediating CML-induced effects on migration, invasion, and stemness in MG63 and U2OS osteosarcoma cells. These cells were pre-treated with a RAGE neutralizing antibody prior to CML treatment. Remarkably, the alterations in EMT-related markers observed in CML-treated cells were effectively reversed by the administration of the RAGE neutralizing antibody. This administration resulted in a significant inhibition of Fibronectin protein expression and an increase in E-cadherin protein expression compared to the CML-treated group (**Figure 6A-C**). Blocking RAGE also significantly mitigated the CML-induced enhancement of cell migration and invasion, as evidenced by **Figure 6D, 6E**. The pre-treatment with the RAGE neutralizing antibody in cells led to a notable reduction in the formation of spheres induced by CML (**Figure 7A, 7B**). This reduction serves as a clear indicator of decreased CSC properties induced by CML treatment. Moreover, the expression of CSC markers, including MRP1, MDR1, CD44, ALDH1A1, and NANOG, exhibited a significant decrease following pre-treatment with the RAGE neutralizing antibody (**Figure 7C-7H**). Collectively, these compelling findings underscore the pivotal role of RAGE in promoting CML-induced stemness in osteosarcoma cells. This insight offers valuable knowledge about the molecular mechanisms underlying the effects mediated by CML/RAGE, particularly in the context of cancer stemness. These effects were closely associated with the activation of a specific signaling pathway known as RAGE/ERK/NFκB (**Figure 8**).

## Discussion

Epidemiological studies have established a strong association between diabetes mellitus and cancer progression 48. However, the underlying molecular mechanisms and factors that contribute to hyperglycemia-induced tumor metastasis remain poorly understood, especially in osteosarcoma. In this study, we demonstrated that hyperglycemia with high serum CML promoted osteosarcoma cell metastasis *in vivo*. While some studies have focused on tumor cell-specific mechanisms to understand the relationship between hyperglycemia and cancer, others have reported that hyperglycemia may not significantly increase primary tumor growth and may even slightly suppress it in certain animal models 49-52. Based on our findings of* in vivo* experiments, we observed that osteosarcoma tumor growth remained unaffected, while hyperglycemia with high serum CML notably augmented tumor metastasis.

Several studies have investigated the role of RAGE in cancer stemness in various types of cancer. For instance, in breast cancer, RAGE has exhibited upregulation in cancer stem cells, correlating with heightened tumorigenicity and metastasis 35. Similarly, in colorectal cancer, RAGE has been shown to be involved in the regulation of stemness-related pathways, including Wnt/β-catenin, Notch, and Hedgehog signaling pathways 53. Moreover, the role for RAGE has been documented in the control of the Wnt/β-catenin pathway, which is critical for maintaining stem cell attributes in glioblastoma 54. However, the precise implications of RAGE in regulating cancer stemness in osteosarcoma remain incompletely elucidated. The present study sheds light on a potential mechanism through which CML augments cancer stem cell properties and metastasis in osteosarcoma cells, achieved via RAGE-mediated activation of the NFκB signaling cascade. The outcomes of this study enhance our comprehension of osteosarcoma biology and pinpoint a potential target for advancing more efficacious therapies against this aggressive bone malignancy. Several preclinical studies have investigated the use of RAGE inhibitors or modulators to assess their impact on tumor growth, metastasis, and response to treatment. Some potential therapeutic targets include small molecules, antibodies, soluble RAGE, and RAGE gene silencing. For instance, TTP488 (Azileragon) is an oral small molecule inhibitor of RAGE that has undergone human clinical trials for Alzheimer's disease 55. TTP488 has also demonstrated the inhibition of metastasis in triple-negative breast cancer (TNBC) cells, suggesting its potential as a therapeutic strategy in TNBC clinical treatment 56. In NSCLC cells, silencing RAGE by siRNA suppresses colony formation, proliferation, migration, and invasion. This may occur through EMT inhibition, impacting crucial signaling pathways like PI3K/AKT and KRAS/RAF-1. Targeting RAGE emerges as a potential therapeutic strategy for NSCLC 57. Alagebrium, also known as ALT7-11, serves as an advanced glycation end product (AGE) cross-link inhibitor. In 2013, a cardiovascular clinical trial (ClinicalTrials.gov Identifier NCT01913301) aimed to evaluate the impact of multi-dose Alagebrium combined with individual exercise on diastolic heart failure. Unfortunately, financial reasons led to the discontinuation of this study. Subsequent trials involved a combination of exercise and 200 mg/day Alagebrium administered for a year among the elderly population. While generally well-tolerated, two individuals experienced gastrointestinal symptoms 58. Despite promising effects observed in diabetic animal models, the translation to humans remains uncertain due to incomplete clinical trials. Presently, there are no ongoing clinical trials involving Alagebrium. In summary, while there is promising preclinical evidence suggesting that targeting RAGE could be a viable strategy for clinical treatment, further research and clinical trials are needed to establish the safety and efficacy of RAGE-directed therapies in human patients with conditions such as osteosarcoma.

The clinical significance of ERK and NFκB in osteosarcoma patients is multifaceted. In osteosarcoma, dysregulation of the ERK pathway has been associated with increased tumor cell proliferation and survival, emphasizing its role in tumor growth 59, 60. Targeting the ERK pathway presents a potential therapeutic strategy to inhibit the progression of osteosarcoma. Sorafenib, a multikinase inhibitor, has been utilized in a multicenter Phase II trial as second- or third-line therapy for patients with unresectable and relapsed high-grade osteosarcoma. In the most challenging scenario of high-grade osteosarcoma, sorafenib demonstrated clinical effectiveness and a well-tolerated toxicity profile 61. Similarly, NFκB, a transcription factor pivotal in inflammation and immune response, is implicated in osteosarcoma tumorigenesis. It has been reported that in osteosarcoma patients, those with negative NFκB and positive PTEN expressin had a significantly higher 5-year survival rate 62. The combined assessment of NFκB and PTEN expression is proposed as a valuable tool for clinically assessing osteosarcoma prognosis, offering a potential new direction for clinical treatment. The cross-talk between ERK and NFκB pathways can impact the behavior of osteosarcoma cells, influencing their response to treatment and overall clinical outcomes. Assessing the expression levels of ERK and NFκB in osteosarcoma tissues may serve as potential prognostic indicators. Elevated activation of these pathways could indicate a more aggressive tumor phenotype, guiding treatment decisions and prognosis assessments. Ongoing research is likely to uncover additional insights into the intricate molecular landscape of osteosarcoma, advancing our understanding and refining therapeutic approaches for improved patient outcomes.

Studies have shown that RAGE can activate AKT signaling pathways, promoting prostate cancer cell proliferation and survival 63. Additionally, RAGE's capability to activate NFκB has been demonstrated through the stimulation of the MAPK and PI3K/Akt pathways 37. Nevertheless, our findings, in contrast to the conventional RAGE-driven AKT activation, demonstrated that CML exposure actually restrained the activation of AKT and GSK3α/β in osteosarcoma cells (Figure 4E). Notably, the previous studies have shown that high AGEs enhance RAGE expression while reducing AKT and COX-2 protein expression in late endothelial progenitor cells (EPCs) 64, and AGEs induce the activation of ERK, JNK, and p38, but inhibit AKT activation in rat vascular smooth muscle cells 65. This hints at the potential involvement of alternative signaling pathways in the NFκB activation induced by CML/RAGE. For example, RAGE has been shown to activate the ERK pathway in various cell types, including endothelial cells, gastric cancer cells, and colorectal cancer cells. In human coronary artery endothelial cells, RAGE triggers the phosphorylation of ERK1/2 and NFκB, leading to the production of ROS 66. In gastric cancer cells, RAGE expression induced by HMGB1 fosters cell proliferation and migration through ERK signaling activation 67. Likewise, HMGB1 released from colorectal cancer cells activates RAGE/ERK/Drp1, leading to cancer cell survival and resistance to chemotherapy 68. However, further investigation is necessary to validate the role of these pathways in CML/RAGE-mediated NFκB activation within osteosarcoma cells.

Activation of RAGE has been shown to trigger the mitogen-activated protein kinase (MAPK) signaling cascades, subsequently releasing and activating NFκB. Initial evidence from studies conducted in rat lung artery smooth muscle cells and neuronal PC12 cells 69 reveals that AGE-albumin treatment induces oxidative stress, activating Ras and ERK, with ERK further stimulating NFκB. Both p38MAPK and ERK are crucial for the induction of NFκB activation by CML adducts/RAGE in human monocytic leukemia cells 70. p38 MAPK activation serves as a mediator for RAGE-induced NFκB-dependent secretion of proinflammatory cytokines in myoblasts. Low doses of S100A8 and A9 have been shown to promote cancer cell growth in a manner dependent on RAGE and the ERK/p38MAPK pathways, with no activation observed in the JNK pathway 71. Activation of the third MAPK pathway, JNK, is involved in RAGE-mediated inflammation. It has been demonstrated in mouse aortic endothelial cells that the induction of vascular cell adhesion molecule-1 (VCAM-1), a marker of vascular inflammation induced by RAGE ligands, can be alleviated through the use of the JNK inhibitor or siRNA targeting JNK 72. Further investigation is needed to validate the specific roles of these MAPK pathways in CML/RAGE-mediated NFκB activation within osteosarcoma cells.

Various inhibitors targeting RAGE have been explored, encompassing anti-RAGE antibodies, soluble RAGE (sRAGE), and inhibitors that target RAGE expression, such as siRNA. Anti-RAGE antibodies have exhibited efficacy in treating conditions involving RAGE overexpression, including cancer 73, 74. These studies have shown that anti-RAGE antibodies can reduce inflammation and improve disease outcomes in animal models. In the present study, we also found that anti-RAGE antibody (neutralizing antibody) could effectively reduce the CML-induced ERK and NFкB signaling, cell mobility, and CSCs properties in osteosarcoma cells. The sRAGE, a truncated version of RAGE, functions as a decoy receptor. By binding to RAGE ligands, it hinders their interaction with cell-bound RAGE 75. This category of inhibitors also encompasses RAGE aptamers and small molecule RAGE inhibitors. These have displayed promising outcomes in preclinical investigations for various RAGE-associated ailments 76, 77. Overall, the development of RAGE inhibitors is an active area of research, and there is potential for these inhibitors to provide therapeutic benefits for various RAGE-associated diseases in the future. However, more exploration is necessary to ascertain their safety and effectiveness through human clinical trials.

While our study provides valuable insights, we also recognize some limitations that warrant consideration. Our study focused mainly on cell lines and animal models, which may not fully represent the real situation of osteosarcoma patients. To enhance the comprehensiveness of our study, further exploration of the serum concentrations of CML or AGEs in osteosarcoma patients is necessary. Additionally, the complexity of the signaling pathways involved in CML/RAGE interactions can be further explored, and our study provides an initial step in this direction. Further investigation is required to understand the potential influence of other microenvironmental factors on CML/RAGE signaling, such as tumor-associated inflammation and the non-tumorous niche. This inflammatory milieu, characterized by the release of cytokines and chemokines, has the potential to both enhance and modulate the CML/RAGE axis. Beyond the immediate tumor site, the non-tumorous niche plays a crucial role. The surrounding tissues, stromal cells, cancer-associated fibroblasts, and immune cells contribute to the overall microenvironment. The presence of CML and the activation of RAGE in these non-tumorous areas may have systemic effects, influencing distant organs and potentially contributing to cancer progression.

In conclusion, the findings of this study propose a potential scenario where hyperglycemia could enhance the metastatic propensity of osteosarcoma cells by upregulating CML and RAGE. Additionally, the application of CML triggered the process of EMT, metastasis, and cancer stemness in osteosarcoma cells. This is accompanied by the concurrent activation of the RAGE/ERK/NFκB signaling pathway and the inhibition of AKT activation. These collective results highlight the prospects of targeting CML/RAGE as viable avenues for prospective therapeutic interventions in the context of osteosarcoma.

## Figures and Tables

**Table 1:** Patient characteristics in osteosarcoma tissue microarray (n = 40).

Characteristics	No.	%
Age		
Age ≥ 60	2	5.0
Age < 60	38	95.0
Median age (range)	26 (11-64)	
Gender		
Male	26	32.5
Female	14	17.5
TNM staging		
T1	7	8.8
T2	33	41.3
N0	39	48.8
N1	1	1.3
Stage		
I	21	26.3
II	18	22.5
IV	1	1.3

T: the size of the original (primary) tumor. N: the number of nearby lymph nodes with cancer.
